# LA PULSE: Evaluating Left Atrial Function Pre- and Post-Atrial Fibrillation Ablation Using PULSEd Field Ablation

**DOI:** 10.3390/jcm14010068

**Published:** 2024-12-26

**Authors:** Noha Mahrous, Florian Blaschke, Doreen Schöppenthau, Gerhard Hindricks, Leif-Hendrik Boldt, Abdul Shokor Parwani

**Affiliations:** 1 Department of Cardiothoracic and Vascular Surgery, Deutsches Herzzentrum Der Charité, 13353 Berlin, Germany; 2 Department of Cardiology, Angiology and Intensive Care Medicine, Deutsches Herzzentrum Der Charité, 13353 Berlin, Germany; 3 DZHK—German Center for Cardiovascular Research, 10785 Berlin, Germany

**Keywords:** atrial fibrillation, pulsed field ablation, left atrial strain, echocardiography, cardiac electrophysiology

## Abstract

**Background**: Atrial fibrillation (AF) is a common cardiac arrhythmia associated with left atrial dysfunction. The impact of pulmonary vein isolation (PVI) using pulsed field ablation (PFA) on left atrial function has not been previously quantified. This study aims to evaluate the effects of PVI using PFA on left atrial function in patients with AF. **Methods**: Thirty-four patients undergoing PVI with PFA between July 2022 and November 2023 were included. The left atrial function was assessed using echocardiography pre-procedure and at 6 months post-procedure. **Results**: The mean age of the patients was 66.5 ± 9.76 years, with 70.6% being male. The cohort included 44% of patients with paroxysmal AF. PVI was successfully achieved in all patients, with a significant improvement in all aspects of left atrial strain at an average of six-month follow-up. The left atrial strain reservoir (LASr) increased from 12.5 ± 5.8% to 21.7 ± 8.1% (*p* < 0.001). Notably, patients with paroxysmal AF exhibited a greater increase in LASr compared to those with persistent AF. Additionally, pre-procedural sinus rhythm was a significant predictor of better LASr outcomes. **Conclusions**: PFA is associated with significant improvement in left atrial reservoir strain, suggesting a positive impact on atrial function. These findings have important implications for the therapeutic management of AF and warrant further research.

## 1. Introduction

Atrial fibrillation (AF) is the most common cardiac arrhythmia, posing a significant global public health challenge. The incidence and prevalence of AF are rising, with an estimated three million new cases worldwide in 2017 alone. Its occurrence increases with age and additional cardiovascular risk factors, highlighting the need for effective and safe management strategies [1,2].

Beyond its direct health impacts, atrial fibrillation (AF) imposes a considerable economic burden on healthcare systems due to the chronic and recurrent nature of the condition. Frequent hospitalizations, often triggered by AF-related complications such as heart failure exacerbations, strokes, or poorly controlled symptoms, represent a significant proportion of these costs [3].

Rhythm control, particularly through atrial fibrillation (AF) ablation, significantly improves patients’ quality of life (QoL) by reducing symptoms. Unlike rate control, rhythm control directly targets the pulmonary triggers of AF, alleviating the physical and mental burden of AF and enhancing overall well-being.

Wokhlu et al. showed that AF ablation provides sustained QoL improvements compared to medications, even in patients with recurrent AF. By reducing AF burden and severity, AF ablation enables patients to regain functional capacity and return to daily activities with fewer limitations [4].

Pulmonary vein isolation (PVI) is the cornerstone of catheter ablation therapy for AF. Traditional PVI techniques, such as radiofrequency and cryoenergy, are not entirely tissue-specific, risking complications by affecting nearby cardiac structures. Pulsed field ablation (PFA) uses biphasic bipolar pulses lasting 2.2 milliseconds at two thousand volts to precisely target heart tissue, potentially reducing risks, shortening procedure times, and lowering pulmonary vein reconnection rates [5,6].

PFA creates lesions with significantly less chronic fibrosis compared to traditional thermal methods. Unlike thermal ablation, which triggers excessive fibrosis through inflammatory responses and microvascular damage, PFA preserves the extracellular matrix and avoids these complications. This unique reparative process is thought to minimize collagen synthesis, possibly leading to improved tissue compliance and better recovery of left atrial mechanical function over time [7].

PFA’s tissue-selective nature is also expected to redefine post-ablation protocols, including a shortened blanking period [8].

Pulsed field ablation (PFA), despite being a relatively new technology, has demonstrated a manageable and relatively short learning curve. Ruwald et al. reported significant reductions in procedural and fluoroscopy times during the implementation phase. Furthermore, the integration of high-density voltage mapping enhanced lesion placement accuracy, supporting efficient workflows and demonstrating the feasibility of PFA in real-world clinical settings [9].

The left atrium’s role in cardiac function is multifaceted: it serves as a reservoir for blood during ventricular systole, acts passively as a conduit for blood transfer, and functions as a booster pump for ventricular filling during diastole, contributing 15–30% of the LV stroke volume [10,11]. These roles are crucial for maintaining efficient cardiac output, particularly in AF patients, where left atrial function is often compromised [12].

Despite its potential, the impact of PFA on left atrial function remains an area of active research. Left atrial strain, especially the left atrial reservoir strain (LASr), is recognized as a key parameter for assessing atrial function, crucial for evaluating cardiac performance in AF patients [10,12]. This study examines the effect of PFA on LASr as well as on the conduit strain LAScd and the contractile strain LASct.

## 2. Materials and Methods

### 2.1. Participant Recruitment

We conducted a retrospective analysis of patients who underwent AF ablation using the FARAPULSE ablation system (Boston Scientific, Marlborough, MA, USA) at our institution in July 2022 and November 2023. Those who consented to a follow-up were subsequently scheduled for an appointment. The follow-up visits included comprehensive echocardiographic evaluations and consultations with a physician. The study’s inclusion criteria included all patients aged between 18 and 99 years who underwent PFA for AF.

Consent for post-interventional follow-up and echocardiographic assessment was mandatory.

### 2.2. Echocardiographic Assessment

**Figure 1:**
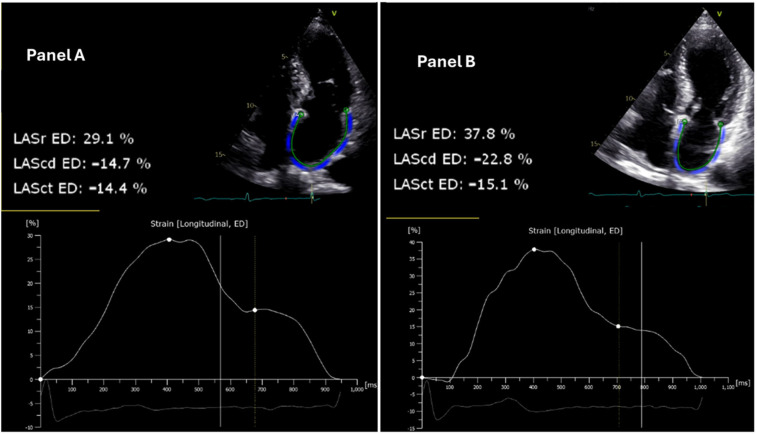
Echocardiographic assessment of left atrial strain reserve in patient 14 before and after pulsed field ablation. The left atrial reservoir strain (LASr), conduit strain (LAScd), and contractile strain (LASct) at the end diastole (ED) are illustrated. (**Panel A**) (left): Pre-ablation two-dimensional speckle-tracking echocardiogram demonstrating a LASr of 29.1%. (**Panel B**) (right): Post-ablation two-dimensional speckle-tracking echocardiogram depicting an increased LASr of 37.8% in the same patient, indicating an improvement in LASr function following the intervention. The strain curves below each echocardiogram reflect the changes in deformation of the left atrial wall over the cardiac cycle, with significant augmentation observed post-ablation.

Pre- and post-interventional echocardiographic assessments were performed using a standard protocol. Left atrial (LA) strain measurements were conducted using Tomtec software Version (LOT): 41.00 and quantified by two-dimensional speckle-tracking echocardiography with TOMTEC-ARENA post-processing software (Tomtec Imaging Systems GmbH, Unterschleißheim, Germany) (Figure 1). LASr, LAScd, and LASct were assessed using 2D speckle-tracking echocardiography (STE). This technique tracks the motion of myocardial speckles in a frame-by-frame manner, allowing for the assessment of myocardial deformation (strain).

The LASr was specifically evaluated during the reservoir phase of the cardiac cycle, which corresponds to the period when the left atrium is filling and expanding during ventricular systole.

In addition to LASr, LAScd and LASct were also evaluated. LAScd was measured during the conduit phase of the cardiac cycle, which corresponds to the passive transfer of blood from the left atrium to the left ventricle during early diastole. LASct was assessed during the booster pump phase, reflecting the active contraction of the left atrium in late diastole.

Rhythm status was recorded during both pre- and post-procedural echocardiographic assessments. This determination was performed at the time of strain measurement to ensure accurate interpretation of left atrial strain parameters.

Strain measurements were recorded as a percentage, reflecting the deformation of the left atrium relative to its resting state.

Patients with poor 2D image quality were excluded. Baseline characteristics and echocardiographic parameters were compared before and after the ablation procedure (Figure 1).

A key requirement was the availability of good quality pre-interventional echocardiographic studies. A modified four-chamber view, optimal for LA strain measurements, was preferred; however, a standard four-chamber view was used when the modified view did not provide adequate image quality. Patients without adequate ECG recordings during pre-interventional echocardiography were excluded, regardless of echocardiographic image quality. Both patients presenting with sinus rhythm and atrial fibrillation were included in the study.

All strain measurements were analyzed by a single observer to ensure consistency and eliminate inter-observer variability. While this approach minimizes variability between observers, intra-observer variability was not formally assessed in this study, representing a potential limitation.

### 2.3. Ablation Procedure

The PFA procedure was performed using the FARAWAVE™ catheter (Boston Scientific, Marlborough, MA, USA) in conjunction with the NAVX™ 3D mapping system (Abbott, Chicago, IL, USA). Pulmonary vein isolation (PVI) was the primary procedural target, aiming to electrically isolate all pulmonary veins. This was the first ablation procedure for all patients. Lesion creation was guided by high-density 3D voltage mapping, and the endpoints of the procedure included confirmation of bidirectional block at all pulmonary veins and non-inducibility of atrial fibrillation, verified through burst atrial pacing at a cycle length of 200 ms.

### 2.4. Statistical Analysis

Due to the exploratory nature of this research, a formal sample size calculation was not performed. Instead, the cohort size was determined by the availability of eligible patients during the study period, balancing practical constraints with the need for comprehensive data collection.

To interpret our findings, 95% confidence intervals were used to indicate the reliability and generalizability of the estimates. Descriptive *p*-values are presented to suggest potential patterns within the data, aligning with the aim of hypothesis generation rather than confirmation. Therefore, these *p*-values should be regarded as preliminary indicators for future research.

**Figure 2:**
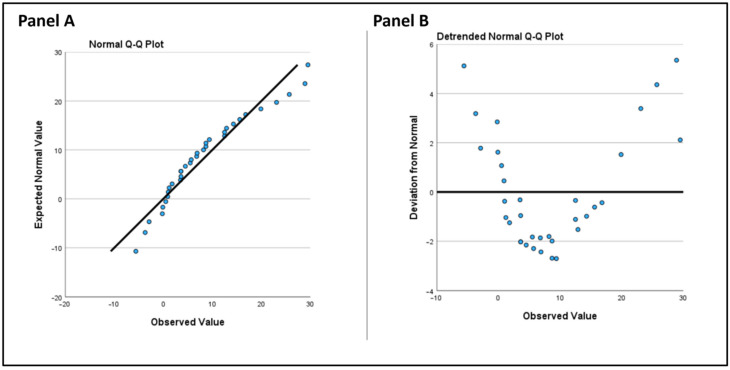
(**Panel A**): Normal QQ plot. The observed values are plotted against expected values from a normal distribution. (**Panel B**): Detrended normal Q-Q plot. This plot displays the deviation of observed values from the expected normal distribution.

For statistical analysis, we utilized IBM SPSS Statistics 29.0 (Armonk, NY, USA). We began by assessing the normality of the distribution for all three parameters, LASr, LAScd, and LASct using the one-sample Kolmogorov-Smirnov test, which is unaffected by sample size. LASr and LASct in our data, comprising 34 observations both pre- and post-intervention (Figure 2), did not significantly deviate from a normal distribution (*p*-values > 0.05), supporting the use of parametric tests for further analysis.

However, LAScd (conduit strain) significantly deviated from a normal distribution post-intervention (*p* = 0.022).

We applied parametric methods to LASr and LASct in our analysis. The paired *t*-test was used as our primary method to compare related samples, specifically the pre- and post-intervention measurements.

Nonetheless, we applied non-parametric test methods to all three variables to account for the possibility of non-normal data in LASr and LASct, using a related-samples Wilcoxon signed rank test.

Non-parametric tests were applied to LASr and LASct as part of a sensitivity analysis to confirm the robustness of the results.

To compare the difference in LASr between paroxysmal and persistent AF groups, an independent samples *t*-test was performed. The difference in LASr was calculated for each group, and the AF type was used as the grouping variable. Cohen’s d was calculated to quantify the magnitude of the difference.

An independent *t*-test was used to evaluate the difference in LASr changes between patients with and without AF recurrence. LASr change was calculated as the difference between post-procedural and pre-procedural LASr values. Patients were grouped based on recurrence status. The analysis included the calculation of mean differences, statistical significance (*p*-value), and effect size (Cohen’s d) to assess the magnitude of the observed differences.

## 3. Results

The study cohort comprised 34 patients undergoing PVI using PFA. The mean age of the patients was 66.5 ± 9.76 years, with a predominance of males (70.6%). Among the participants, 44% had paroxysmal AF. The mean Body Mass Index (BMI) was recorded at 26.7 ± 5.0 kg/m^2^, and the mean CHA2DS2-VASc score was 2.5 ± 1.5 (Table 1).

Echocardiographic evaluations indicated a left ventricular ejection fraction (LVEF) of 54 ± 10%. The mean tricuspid annular plane systolic excursion (TAPSE) was 18.9 ± 2.0 mm. The left ventricular end-diastolic volume (LVEDV) averaged 117 ± 36 mL, and the left atrial volume index (LAVI) was 45.7 ± 17 mL/m^2^.

**Figure 3:**
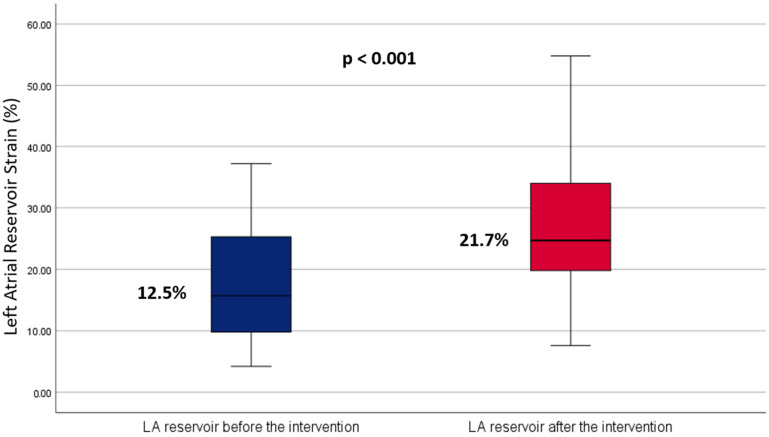
Boxplot comparing left atrial reservoir strain before and after ablation. The blue boxplot represents pre-intervention measurements, while the red boxplot shows post-intervention measurements. This visual comparison highlights the central tendency and variability of the strain measurements before and after the ablation procedure.

Pre-ablation, the mean LASr was 12.5 ± 5.8%. This increased significantly to 21.7 ± 8.1% post-ablation (*p* < 0.001), with a mean difference of −8.33 (95% CI: −11.51 to −5.15, *p* < 0.001) and a large effect size (Cohen’s d = 0.915) (Figure 3). Subgroup analyses showed that patients with paroxysmal AF experienced an increase in LASr from 24.2 ± 7.9% to 31.4 ± 10% (*p* < 0.001) (Table 2). Meanwhile, those with persistent AF showed an increase in LASr from 12.5 ± 5.8% to 21.7 ± 8.1% *p* < 0.001.

There was, however, no statistically significant difference in LASr improvement between paroxysmal and persistent AF groups (*p* = 0.528). The effect size was small (Cohen’s d = −0.22), indicating a minimal practical difference.

The improvement in LASr was accompanied by corresponding improvements in other echocardiographic parameters, including reductions in left atrial volume. The mean LAVI decreased from 45.7 ± 17 mL/m^2^ to 38.2 ± 15 mL/m^2^ post-ablation (*p* < 0.01), indicating a reduction in atrial size and potentially less atrial remodeling.

Although gender and type of AF did not predict changes individually (gender, *p* = 0.791; type of AF, *p* = 0.530), a significant interaction (*p* = 0.048) suggested a differential effect of PFA based on these patient characteristics. However, the overall model did not reach statistical significance (*p* = 0.920).

Notably, pre-procedural cardiac rhythm was found to significantly predict improvement in LASr (*p* = 0.046), with better outcomes observed for patients in sinus rhythm before the procedure.

Additionally, a significant increase in contractile strain after the intervention was observed (mean difference = 3.86 ± 6.89, *p* = 0.003) as well as a significant improvement in conduit strain (LAScd) (*p* < 0.001).

The mean LASr change was 9.40 ± 9.51% for patients without recurrence and 4.85 ± 7.08% for those with recurrence. Although patients without recurrence demonstrated a greater improvement in LASr, the difference was not statistically significant (t(32) = 1.247, *p* = 0.222). The effect size, as measured by Cohen’s d, was 0.50, indicating a moderate but non-significant effect. 

## 4. Discussion

Pulsed field ablation is distinguished by its superior safety profile, tissue selectivity, reduced rates of pulmonary vein reconnection, and shortened procedure times [5,6].

While RF and cryoablation have shown significant improvements in left atrial ejection fraction (LAEF) due to reverse remodeling, they are associated with fibrotic remodeling and a higher risk of impaired atrial compliance [13,14].

In contrast, PFA’s tissue-selective mechanism spares surrounding structures and minimizes chronic fibrosis, preserving LA compliance and promoting sustained functional recovery. Furthermore, PFA may reduce the risk of LAEF decline, which has been correlated with arrhythmia recurrence in other modalities. These findings position PFA as a promising alternative to thermal ablation techniques, with the potential to optimize both safety and long-term functional outcomes.

The assessment of left atrial contractile strain serves as a critical surrogate for evaluating left atrial function, reverse remodeling, and cardiac diastolic function. Previous studies have demonstrated improvements in LASr following AF ablation, primarily using traditional energy sources such as radiofrequency and cryoenergy [13,14].

Our study extends these findings by demonstrating that PFA is associated with significant improvements in all left atrial strain aspects in patients with symptomatic AF. The considerable increase in left atrial strain underscores PFA’s potential to enhance cardiac function.

### 4.1. Improvement in Left Atrial Strain

The statistically significant increase in LASr post-PFA highlights the efficacy of this ablation technique in enhancing atrial function. This is particularly noteworthy given the crucial role of the left atrium in overall cardiac performance, especially in AF patients where compromised atrial function is common.

A recently published small study reported similar results, demonstrating improvements in left atrial strain, active emptying fraction, and expansion index at three months post-ablation. These results are in concordance with our findings, reinforcing the potential of PFA to enhance atrial mechanical function [15].

While our findings suggest a significant improvement in LASr following PFA, it is important to note that in the absence of a control group, these results cannot be solely attributed to the specific energy type. The observed effects may also be influenced by the reduction in AF burden or other non-specific benefits of rhythm control achieved through catheter ablation.

In our subgroup analysis, both paroxysmal and persistent AF patients demonstrated significant improvements in LASr following PFA. However, the magnitude of improvement did not differ significantly between the two groups. Given that persistent AF is typically associated with worse clinical outcomes and higher rates of major cardiac events compared to paroxysmal AF [16], the observed improvement in LASr regardless of AF type suggests that PFA remains a viable and effective treatment option even for patients with persistent AF.

The significant improvement in left atrial strain following PFA suggests a dual benefit of this procedure: not only targeting the arrhythmogenic substrate but also promoting better atrial mechanical function. This could translate into improved clinical outcomes for AF patients, potentially reducing the burden of this arrhythmia on the healthcare system.

The interaction between gender and AF type in influencing LASr changes post-PFA is an intriguing aspect of our results. Although our model did not demonstrate strong overall predictive power, this interaction may be attributed to the small sample size or patient-specific factors influencing the efficacy of PFA. These observations warrant further investigation to better tailor PFA to individual patient profiles and optimize outcomes.

Furthermore, the improvement in left atrial strain was not limited to the reservoir function (LASr) but also extended to conduit strain (LAScd) and contractile strain (LASct), indicating enhanced left atrial performance across all functional phases.

The observed non-normal distribution of LAScd post-intervention is likely due to the small sample size, which may have influenced the statistical assessment of normality.

Our analysis indicates a trend toward greater improvement in LASr among patients without AF recurrence compared to those with recurrence; however, this difference was not statistically significant. This lack of statistical significance could be attributed to the limited sample size. Future studies with larger cohorts are needed to confirm these observations.

### 4.2. Potential Mechanisms

a. Tissue selectivity:

PFA’s mechanism of action involves creating pores in the myocyte cell membrane through irreversible electroporation, leading to targeted cell death [5]. This selective tissue ablation spares surrounding non-cardiac tissues, reducing the risk of collateral damage. Studies have shown that PFA induces large acute late gadolinium enhancement (LGE) without microvascular damage or intramural hemorrhage. This selective ablation contributes to improved atrial mechanical function and reduced complications, making PFA a promising alternative to thermal ablation techniques.

b. Anti-remodeling effect:

A study comparing PFA with thermal ablation found that PFA prevented chronic atrial fibrotic changes and restrictive mechanics, with most LGE lesions disappearing in the chronic stage, indicating a specific reparative process [7]. These long-term benefits highlight PFA’s potential to improve patient outcomes and reduce the burden of AF on the healthcare system.

Recent evidence also suggests that the traditional 3-month blanking period may not fully apply to PFA due to its lower inflammatory response, with a shortened blanking period of 1 month proposed based on its predictive value for late arrhythmia recurrence. These findings highlight PFA’s potential to improve procedural safety, preserve atrial function, and redefine post-ablation management protocols [8].

### 4.3. Implications of the Findings

Our study has demonstrated that PFA is associated with a statistically significant improvement in all aspects of left atrial strain (LASr, LAScd, and LASct), an indicator of enhanced atrial function. This finding can have significant implications, as improved left atrial strain post-ablation could translate into better overall cardiac function for patients, potentially leading to reduced symptoms, improved exercise capacity, and quality of life. Moreover, the observed improvement in left atrial strain may also have prognostic implications, as previous studies have linked it to lower rates of atrial fibrillation recurrence [10]. Hence, our results suggest that PFA may not only serve as an effective treatment for rhythm control but also confer long-term structural benefits to the atrium, which could influence future AF management strategies.

### 4.4. Study Limitations

A key limitation of our study is the small sample size and the short follow-up period. While our results are statistically significant, larger studies with longer follow-up are needed to confirm these findings and assess the long-term impact of PFA on atrial function. Additionally, our study was conducted at a single center, which may limit the generalizability of the findings. Future multicenter studies could provide a more comprehensive understanding of the effects of PFA on left atrial function.

Furthermore, the ablations were performed using the FARAPULSE ablation system (Boston Scientific, Marlborough, MA, USA), which is not representative of all systems using pulsed field ablation. Additional studies using other systems for pulsed field ablation are needed to generalize these findings.

Another limitation of our study is the absence of AF burden estimation in the patient cohort. As AF burden increasingly emerges as a critical determinant of procedural success and long-term outcomes, its quantification could have provided valuable insights into the relationship between recurrence, LASr changes, and atrial remodeling. Incorporating AF burden in future studies would allow for a more comprehensive evaluation of the factors influencing left atrial functional recovery and overall efficacy of AF ablation.

Another limitation was the variability in echocardiographic image quality, common in clinical settings. Although we aimed to use a modified four-chamber view optimal for left atrial strain measurements, this was not always feasible, and in such cases, a standard four-chamber view was used.

### 4.5. Future Research Directions

Future research should focus on exploring the long-term outcomes of PFA on LA function and examining the impact of this procedure on patients’ quality of life and long-term rhythm control as well as exploring patient-specific predictors of response to PFA.

## 5. Conclusions

In conclusion, our explorative study provides evidence that pulsed field ablation is associated with significant improvement in left atrial reservoir strain among patients with atrial fibrillation. Given the study’s observational design and considering these promising results, further randomized trials are needed to elucidate the relationships between pulsed field ablation and improved atrial function in patients with atrial fibrillation and to solidify PFA’s place in the AF management continuum.

## Figures and Tables

**Table 1:** Baseline characteristics of the study cohort.

Baseline Characteristics (*n* = 34)	
Age (years)	66.5 ± 9.76
Women	10 (29.4%)
Paroxysmal	15 (44%)
TAPSE in mm	18.9 ± 2
BMI (kg/m^2^)	26.7 ± 5
Left ventricular ejection fraction (%)	54 ± 10
CHA2 DS2-VASc score	2.5 ± 1.5
LVEDV	117 ± 36
LAVI	45.7 ± 17

This table presents the baseline demographic and clinical characteristics of the 34 patients included in the study. The parameters include age, gender distribution, type of atrial fibrillation (paroxysmal or persistent), tricuspid annular plane systolic excursion (TAPSE), body mass index (B MI), left ventricular ejection fraction (LVEF), CHA2DS2-VASc score, left ventricular end-diastolic volume (LVEDV), and left atrial volume index (LAVI). Values are expressed as mean ± standard deviation (SD) or as absolute numbers with percentages in parentheses.

**Table 2:** Left atrial strain reservoir (LASr) in paroxysmal and persistent atrial fibrillation pre- and post-intervention.

	Left Atrial Strain Reservoir Pre-Interventional (Mean ± SD)	Left Atrial Strain Reservoir Post-Interventional (Mean ± SD)	*p*-Value
Paroxysmal AF	24.2 ± 7.9	31.4 ± 10	0.015
Persistent AF	12.5 ± 5.8	21.7 ± 8.1	<0.001

This table compares the left atrial strain reservoir (LASr) measurements before and after pulsed field ablation (PFA) in patients with paroxysmal and persistent atrial fibrillation. The table shows mean ± SD values for pre-interventional and post-interventional LASr, as well as the *p* value.

## Data Availability

The datasets generated and/or analyzed during the current study are not publicly available due to privacy or ethical restrictions but are available from the corresponding author on request. De-identified data can be made available while maintaining confidentiality.
